# Mass Screening of SARS-CoV-2 With Rapid Antigen Tests in a Receding Omicron Wave: Population-Based Survey for Epidemiologic Evaluation

**DOI:** 10.2196/40175

**Published:** 2022-11-09

**Authors:** Tsz Ho Kwan, Ngai Sze Wong, Chin Pok Chan, Eng Kiong Yeoh, Samuel Yeung-shan Wong, Shui Shan Lee

**Affiliations:** 1 Stanley Ho Centre for Emerging Infectious Diseases The Chinese University of Hong Kong Shatin Hong Kong; 2 Jockey Club School of Public Health and Primary Care The Chinese University of Hong Kong Shatin Hong Kong

**Keywords:** COVID-19, SARS-CoV-2 antigen testing, COVID-19 vaccine, mass screening, antigen test, epidemiology, Omicron, Hong Kong, public health, outbreak, epidemic, screening, transmission, online, vaccination, vaccines, surveillance

## Abstract

**Background:**

The COVID-19 Omicron BA.2 epidemic wave in Hong Kong peaked in the first quarter of 2022. Following the implementation of stringent public health measures, the daily number of reported cases fell from over 50,000 to below 2000. Although outbreaks steadily receded, the government rolled out a 3-day “voluntary universal rapid testing” campaign to invite all citizens to self-perform a rapid antigen test (RAT) daily to identify undetected prevalent infections.

**Objective:**

This study aimed to evaluate the uptake and results of RAT mass screening to estimate the population’s residual epidemic burden and assess the risk of further transmission.

**Methods:**

A cross-sectional study comprising an open web-based population-based survey was conducted a week after the RAT campaign. Participants were asked to report their COVID-19 vaccination and infection history and the RAT performance and test result during the period. They were also invited to report their coliving individuals’ test performance and results. Reasons for nonuptake were enquired. Testing and positive rates were age-adjusted. Determinants of undergoing RAT were identified using univariable and multivariable logistic regression models.

**Results:**

In total, particulars from 21,769 individuals were reported by 8338 participants. The overall age-adjusted testing rate was 74.94% (95% CI 73.71%-76.18%), with over 80% of participants in the age groups between 45-84 years having self-performed RAT during the campaign period. After age-adjustment, 1.03% (95% CI 0.86%-1.21%) of participants tested positive. The positive rates in the age groups between 20-29 years and >84 years exceeded 2%. Taking into account the positive rate and 5819 reported cases during the period, the cases identified in the campaign might account for 7.65% (95% CI 6.47%-9.14%) of all infections. Testers were more likely to be female, older, not previously diagnosed with COVID-19, and have received COVID-19 vaccination. Adjusting for the number of household members, those living with a child aged <12 years and whose household members were also tested were more likely to have self-performed an RAT. Main reasons for not performing an RAT included the absence of symptoms (598/1108, 53.97%), disbelief of the appropriateness of the campaign as an antiepidemic measure (355/1108, 32.04%), and a recent COVID-19 diagnosis (332/1108, 29.96%).

**Conclusions:**

The residual population burden remained substantial in spite of the clear evidence of a receding epidemic wave. Despite caution in generalization to the Hong Kong population, the high participation rate in mass screening indicated that the voluntary RAT was well accepted, making it a feasible option for implementation as a complementary means of public health surveillance.

## Introduction

Worldwide, SARS-CoV-2 transmissions are characterized by repeated outbreak waves of different intensities and amplitudes. In 2020 and 2021, three waves of SARS-CoV-2 transmission in Hong Kong, a densely populated Asia-Pacific city, were brought under control with stringent public health and social measures, comprising case detection, contract tracing, isolation of infected persons, quarantine of close contacts, and widely accessible polymerase chain reaction (PCR) tests in health care facilities and community centers [1]. Social and mobility restrictions were imposed once community transmission had been detected, thereby shifting the epidemic burden to other less restricted exposure settings [2]. By late 2021, no local transmissions were detected for almost 3 months. This enviable record was broken when the first cases of Omicron BA.2 infections became detected in the community, causing a superspreading event [3]. Despite reimposing restrictions to social activities and mobility in anticipation of increased social mix in the Lunar New Year holiday period that followed, Hong Kong was hard hit by the Omicron BA.2 epidemic in February and March 2022, with over 50,000 cases reported daily at its peak [4]. Although the epidemic was receding, the government rolled out a 3-day “voluntary universal rapid testing” campaign between April 8-10, 2022, during which citizens were invited to self-perform a rapid antigen test (RAT) daily in the absence of any lockdown policies while other social distancing measures remained in place [5]. Antiepidemic service bags containing, inter alia, 20 RAT kits were distributed to all households across the city a week in advance. A web-based declaration system was in place to facilitate the statutory reporting of positive cases within 24 hours for issuing isolation and quarantine orders. In the week prior to screening, the daily number of COVID-19 cases reported had decreased to below 5000, and a downward trend was observed [6].

Mass screening is an uncommon control strategy for COVID-19, and only limited studies on its application have been published [7,8], although it has been suggested for developing an exit strategy [9]. Guangzhou’s mass-screening exercise in 2021, along with the isolation and city border control policies, had contributed to the suppression of the epidemic in 6 months [8]. A modeling study in Slovakia showed that after the mass-testing campaign, the prevalence could be reduced by 70% [10]. Another modeling study in France demonstrated that, on average, the RAT-based mass-testing campaign could reduce daily incidence by up to 30% [11]. However, these campaigns do not necessarily contribute to the reduction of mortality [12]. A web-based survey conducted in United States showed that, for voluntary testing without a stipulated mass-testing period, the reasons for self-testing included potential exposure and the presence of symptoms [13]. The uptake rate also varied across geographic regions and age groups. The role of RAT is not limited to case detection but also surveillance, particularly in places adopting the “living with the virus” policy, thereby informing public health policies [14]. Against these backgrounds, we conducted a population-based survey on the uptake and results of RAT mass screening to estimate the population’s residual SARS-CoV-2 burden and assess the risk of further transmission in the territory.

## Methods

### Study Design

This was a cross-sectional study. Eligible participants were Hong Kong residents aged ≥18 years whose households had received an antiepidemic service bag distributed by the government. A bilingual (Chinese and English versions), open, self-administered, web-based, and population-based survey was designed, covering demographics (age, sex, and residing district); COVID-19 vaccination history (type, date, and dose of the last vaccination received); COVID-19 infection history; signs and symptoms; RAT performed during the 3 campaign days with result; post–positive result actions (reporting to the government’s web-based declaration system, seeking treatment, and isolation); and the number of coliving individuals. Participants could opt to report their coliving individuals’ age, sex, RAT performed and result during the campaign period, and their relationships, up to 5 persons. Due to the simplistic nature of this study, the items were not randomized. Adaptive questioning was used on the same page to display questions relating to COVID-19 vaccination history, details about RAT history during the campaign period, and particulars about the coliving individuals. For those who did not undergo an RAT, they were asked to select at least one of the following reasons for not doing so: recent diagnosis, recently tested, regular testing as part of work requirement, no appropriate time and environment, avoiding isolation if tested positive, avoiding compulsory declaration if tested positive, avoiding sampling discomfort, no confidence to self-test, no symptoms, not worried about getting infected, not believing the campaign was an appropriate antiepidemic measure, and others. There were at most 16 questions for each participant, and a maximum of 5 questions for each coliving individual. No personal identifiers were collected. The survey was tested and refined before fielding. After completing the survey, participants were invited to share their location using the HTML5 Geolocation Application Programming Interface if they were at home or in the workplace. Coordinates outside the territory of Hong Kong were removed.

### Subjects and Recruitment

Web and newspapers advertisements were placed to recruit Hong Kong residents to join as participants. All responses were collected through the bespoke website designed for this web-based survey. The completion of the survey by participants was voluntary. Completeness checking was done using JavaScript before submission. Incomplete responses were not collected. No data were excluded due to atypical time stamps because of the simplistic nature of the survey. An anonymous session identifier was set in the cookies, and the IP addresses of participants were collected. Duplicate entries of the same session identifier were removed.

### Ethics Approval

The study data collected were anonymous. Web-based informed consents were obtained before participants filled out the questionnaire. No incentives were offered upon the completion of the study. This study was approved by the Survey and Behavioural Research Ethics Committee of The Chinese University of Hong Kong (SBRE-21-0685). The conduct of the study was in compliance with the Declaration of Helsinki.

### Statistical Analysis

Determinants of undergoing an RAT during the campaign period were identified using univariable and multivariable logistic regression models. Testing rate and positive rate were determined by aggregating both study participants and their coliving participants. The Wilson score method was used to calculate the 95% CI of age-specific testing rate [15]. The testing rate and positive rate were age-adjusted by groups defined by 5-year windows, except those aged ≥85 years were grouped together, using the provisional figures published by the Census and Statistics Department from the 2021 Population Census [16]. The 95% CI of the directly standardized testing rate was computed using the Byar method [17]. The number of prevalent infections in the territory during the campaign period was estimated by multiplying the population size by the age-adjusted positive rate with the 95% CI. Maps were drawn with the QGIS platform (QGIS Development Team) using 2019 District Council Constituency Areas as the spatial unit. There was a total of 452 District Council Constituency Areas, each of which normally containing about 16,599 residents [18]. Secondary outcomes, including determinants of prior COVID-19 diagnosis and reasons for not getting tested, were evaluated using chi-square test and Mann-Whitney *U* test for categorical and continuous predictors, respectively. Multivariable logistic regression analysis was performed by including variables with a *P* value of <.05 in the univariable analyses. All statistical analyses were conducted in R statistical software (R Foundation for Statistical Computing). Reporting in this manuscript follows the Checklist for Reporting Results of Internet E-Surveys [19].

## Results

**Figure 1:**
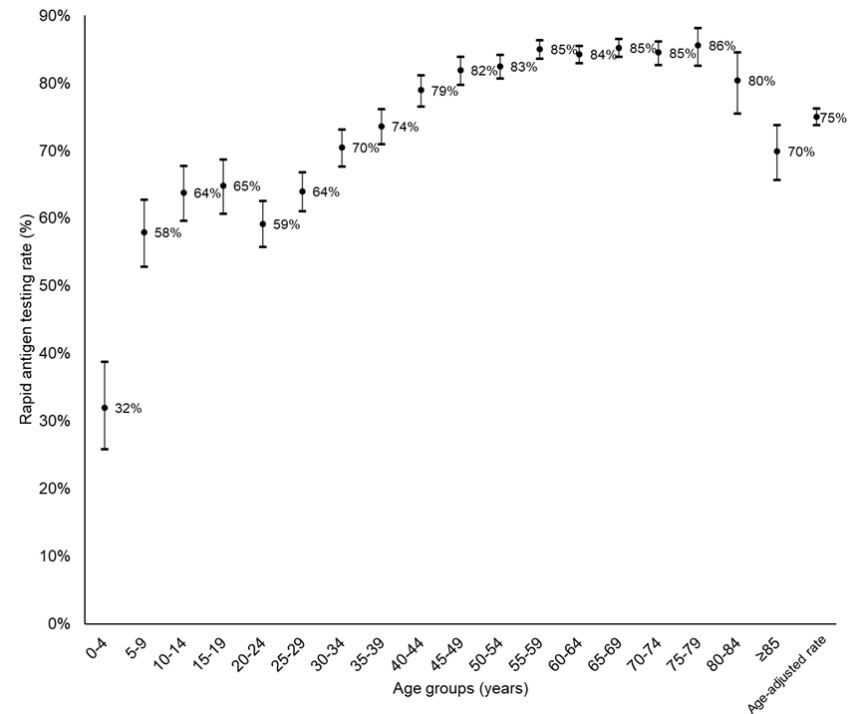
Age-specific and age-adjusted rapid antigen testing rates (dots) and 95% CIs (error bars).

In total, 8759 responses were collected between April 17-25, 2022, of which 8338 were analyzed after removing duplicate entries. Of the 8338 participants, the median age was 61 (IQR 53-67) years, and 38.89% (n=3243) were male (Table 1). In all, 16.89% (n=1408) reported at least one episode of previous COVID-19 diagnosis. Almost all (8086/8314, 97.26%) participants have received at least one dose of COVID-19 vaccine, with 81.48% (6774/8314) having 3 doses or more. The distribution of the types of vaccine received for the last dose was similar (BNT162b2 by Pfizer-BioNTech: 4566/8105, 56.34%; CoronaVac by Sinovac: 3522/8015, 43.45%; and others: 17/8105, 0.21%). The median number of coliving individuals was 2 (IQR 1-3), totaling 15,243 persons, of whom the particulars of 13,431 (88.11%) household members were complete for analysis. Combining both index respondents and coliving household members (N=21,769), the overall median age was 56 (IQR 38-65) years, and the overall crude RAT self-screening rate was 78.53% (n=17,096), with age-specific rates of over 80% in the age groups between 45-84 years. The overall age-adjusted testing rate was 74.94% (95% CI 73.71%-76.18%; Figure 1). Although geographical variation of the proportion of households who performed the RAT was observed, there were no significant differences among spatial units (n=6949; *P*>.99 by chi-square test; Multimedia Appendix 1).

Among index participants, having performed an RAT was associated with one’s sex (reference: female; adjusted odds ratio [aOR] 0.76, 95% CI 0.67-0.87; *P*=.001), older age in years (aOR 1.03, 95% CI 1.03-1.04; *P*<.001), a previous COVID-19 diagnosis (aOR 0.42, 95% CI 0.37-0.49; *P*<.001), and vaccination history (aOR 2.03, 95% CI 1.46-2.78; *P*<.001; Table 2). Among those vaccinated, the number of doses (aOR 1.80, 95% CI 1.58-2.06; *P*<.001) and type of the last vaccine dose received (Sinovac compared to BioNTech: aOR 2.28, 95% CI 1.96-2.66; *P*<.001) were associated with testing during the campaign period. Taking factors regarding coliving individuals into account, after adjusting for the number of household members, household members having been tested (*P*<.001) and living with a child or children aged <12 years (*P*=.002) were additionally associated with RAT performance during the campaign period.

**Figure 2:**
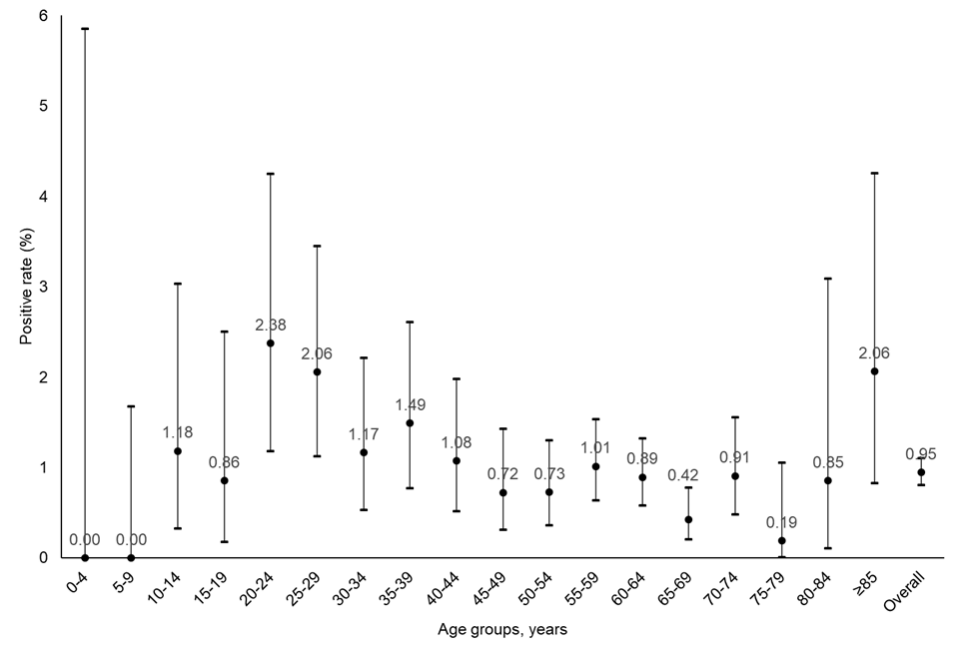
Age-specific and age-adjusted positive rates (dots) and 95% CIs (error bars).

Among the reasons (n=1108) for not getting tested, the 3 most common ones were not having symptoms (n=598, 53.97%), not believing the campaign was an appropriate antiepidemic measure (n=355, 32.04%), and a recent diagnosis (n=332, 29.96%; Table 3). Factors associated with prior diagnosis included not living alone (odds ratio [OR] 1.49, 95% CI 1.23-1.79; *P*<.001), especially with those aged <12 years (OR 1.32, 95% CI 1.08-1.62; *P*=.007); age (*P*<.001, by Mann-Whitney *U* test); and not having been vaccinated with at least 2 doses (OR 2.11, 95% CI 1.68-2.66; *P*<.001). The multivariable logistic regression showed that age in years (aOR 0.98, 95% CI 0.97-0.98; *P*<.001), not living alone (aOR 1.42, 95% CI 1.18-1.72; *P*<.001), and receiving less than 2 vaccination doses (aOR 2.05, 95% CI 1.60-2.60; *P*<.001) were significantly associated with prior diagnosis. The untested respondents who did not believe the campaign was an appropriate antiepidemic measure were more likely to be of younger age in years (aOR 0.99, 95% CI 0.98-0.99; *P*=.001), male (aOR 1.70, 95% CI 1.31-2.20; *P*<.001), and unvaccinated (aOR 1.95, 95% CI 1.12-3.39; *P*=.02). Notably, only a small proportion of participants reported being not confident to perform a self-test (n=32, 2.89%) and not having time and an appropriate environment to test (n=8, 0.72%). Separately, the crude positive rate among participants and including their coliving individuals was 0.62% (45/7226) and 1.19% (117/9870), respectively. Age-specific positive rates exceeded 2% in the population groups between 20-29 years and those aged >85 years (Figure 2). After adjusting for the age structure of the population, 1.03% (95% CI 0.86%-1.21%) of the population could have tested positive during the campaign period. It can be inferred that 76,039 (95% CI 63,663-89,947) persons in the 7.4 million population could have tested positive during the 3 days. In term of compliance, of the 45 participants reporting a positive RAT result, 62% (n=28) had declared to the government and 73% (n=33) self-isolated. Some 64% (29/45) sought treatments, including self-medications reported in a majority (22/29, 76%) of participants.

**Table 1:** Demographics and COVID-19–related histories of participants and their household members.

Characteristic		Participants	Participants’ household members
Sex, male (participants: n=8338; participants’ household members: n=13,431), n (%)		3243 (38.89)	5593 (41.64)
Age (years; participants: n=8338; participants’ household members: n=13,431), median (IQR)		61 (53-67)	50 (30-63)
Performed an RAT^a^ during the campaign period (participants: n=8338; participants’ household members: n=13,431), n (%)		7226 (86.66)	9870 (73.49)
Performed an RAT more than once during the campaign period (n=7226), n (%)		6258 (86.6)	N/A^b^
Tested positive during the campaign period (participants: n=7226; participants’ household members: n=9870), n (%)		45 (0.62)	117 (1.19)
Previous COVID-19 diagnosis (n=8338), n (%)		1408 (16.89)	N/A
**Number of COVID-19 vaccines received (n=8314), n (%)**			
	0	228 (2.74)	N/A
	1	122 (1.47)	N/A
	2	1190 (14.31)	N/A
	3	6425 (77.28)	N/A
	4	349 (4.2)	N/A
**Type of the last COVID-19 vaccine received (n=8105), n (%)**			
	BNT162b2 by BioNTech	4566 (56.34)	N/A
	CoronaVac by Sinovac	3522 (43.45)	N/A
	Others	17 (0.21)	N/A

^a^RAT: rapid antigen test.

^b^N/A: not applicable.

**Table 2:** Factors associated with performing a rapid antigen test during the campaign period.

Factor	Model 1	*P* value	Model 2	*P* value	Model 3	*P* value
Sex, male (reference: female), aOR^a^ (95% CI)	0.76 (0.67-0.87)	<.001	0.75 (0.66-0.86)	<.001	0.75 (0.62-0.89)	.002
Age (years), aOR (95% CI)	1.03 (1.03-1.04)	<.001	1.02 (1.02-1.03)	<.001	1.02 (1.01-1.03)	<.001
Previous diagnosis of COVID-19, aOR (95% CI)	0.42 (0.37-0.49)	<.001	0.47 (0.41-0.56)	<.001	0.45 (0.37-0.56)	<.001
Vaccinated for at least one dose against COVID-19, aOR (95% CI)	2.03 (1.46-2.78)	<.001	N/A^b^	N/A	N/A	N/A
Received Sinovac COVID-19 vaccine (reference: BioNTech vaccine), aOR (95% CI)	N/A	N/A	2.28 (1.96-2.66)	<.001	1.97 (1.62-2.39)	<.001
Number of doses of COVID-19 vaccination, aOR (95% CI)	N/A	N/A	1.80 (1.58-2.06)	<.001	2.02 (1.62-2.39)	<.001
Number of household members, aOR (95% CI)	N/A	N/A	N/A	N/A	0.53 (0.48-0.58)	<.001
Coliving with a person aged <12 years, aOR (95% CI)	N/A	N/A	N/A	N/A	1.81 (1.26-2.61)	.002
Any of the coliving individuals having been tested during the campaign, aOR (95% CI)	N/A	N/A	N/A	N/A	7.28 (6.36-8.36)	<.001
AIC^c^	6208		5739		3336	

^a^aOR: adjusted odds ratio.

^b^N/A: not applicable.

^c^AIC: Akaike information criterion.

**Table 3:** Reasons for not having performed rapid antigen testing during the campaign period (n=1108).

Reason	Participant, n (%)
Not necessary because I have been diagnosed recently	332 (29.96)
Unwilling to repeat because I have tested recently	181 (16.34)
Doing testing regularly as part of work requirement, so would not want to do additional tests	95 (8.57)
Did not have the time and the appropriate environment to do the test	8 (0.72)
To avoid isolation due to a positive result	97 (8.75)
To avoid declaration of a positive result to the government	58 (5.23)
To avoid discomfort caused by swab collection	60 (5.52)
Not confident to perform a self-test	32 (2.89)
Not having symptoms	598 (53.97)
Not worried about getting infected	204 (18.41)
Not believing that “voluntary universal rapid testing” campaign is an appropriate antiepidemic measure	355 (32.04)
Other reasons	95 (8.57)

## Discussion

### Principal Findings

Participation is key to a voluntary mass-screening campaign for SARS-CoV-2 infection, the coverage of which would not be known in the absence of an accompanying surveillance mechanism. An evaluation of the Hong Kong campaign was made possible through a separately conducted ensuing population-based web-based survey. The geographical diversity of the participants in this study supported that a diverse sample population had been recruited. The proportion of participants reporting previous COVID-19 diagnosis corresponded well with that recorded (about 16%) in the published government statistics [6]. Although participation in mass screening was voluntary, three-quarters of respondents in our population-based survey had undergone the testing at least once during the 3-day period, confirming the feasibility of implementing self-RAT screening as a complementary means of public health surveillance.

Our survey results showed that those participating in the RAT campaign were more likely to be older, female, and vaccinated against COVID-19. These characteristics were similar to participants in a previous PCR-based voluntary screening campaign in 2020, which showed that participation was associated with perceived efficacy of the campaign in controlling the epidemic, perceived susceptibility to COVID-19, and their trust of the government [20]. About one-third of nontesters did not believe the RAT campaign could control the epidemic. Such a low perceived efficacy might have prevented some citizens from participating in the campaign. The mandatory reporting of positive results had lowered the willingness of a certain proportion of people to participate. The higher odds of being vaccinated against COVID-19 and receiving more doses among testers could be a result of one’s perceived susceptibility. Trust in the government that the policies are efficacious in controlling the epidemic could contribute to their engagement in vaccination and screening [20]. On the contrary, distrust in the government could also contribute to self-regulation to prevent infection and protect one’s own interests, leading to passive compliance with antipandemic measures [21]. Separately, in the recent epidemic waves in Hong Kong, children were more likely to be asymptomatic and be infected through household transmissions rather than exogenous acquisition from schools [22], which might have prompted household members living with children to be tested to prevent transmission to the younger members if they tested positive.

Differentiating the epidemic situation between the time of the 2020 PCR-based screening campaign and the 2022 RAT-based one, a greater proportion of population had already been infected prior to the latter campaign, which may have affected the participation rate as some residents may not consider it necessary to undergo testing. The presence of symptoms was one of the indicators of SARS-CoV-2 infection, which prompts one to have testing performed [23]. This testing process was educated publicly to encourage people to get tested when they are presented with symptoms; on the other hand, people without symptoms may not be interested in taking the RAT. Although the figures in the previous study cannot be directly compared with findings from this study due to methodological differences, it is worth noting that the participation rate of the previous PCR-based screening campaign was only 47%, and one-quarter of nonparticipants noted they did not have time for screening [20]. With three-quarters of respondents having participated in the self-RAT screening and just 1% concerned about spending time on it, the convenience and acceptability of an RAT-based voluntary screening campaign over a PCR-based one was highlighted.

As the official number of locally reported cases was just 5819 during the campaign period [6], the reported cases might have accounted for about 7.65% (95% CI 6.47%-9.14%) of all infections given the 1% positive rate; the rest being not reported despite statutory requirement or not detected because of either the low sensitivity in picking up early infections or that no screening had been performed. As only 62% participants who tested positive declared their results to the government, the number of reported cases could be underestimated. Since a higher proportion self-isolated after receiving a positive result, they were willing to take precaution to prevent onward transmissions in the community, although they did not declare the results to the authority. The high mobility of the younger working population and older adults were not reduced much during the epidemic, which predisposed them to the risk of infection [24]. Evidently, the Omicron wave has rapidly receded after over a million people have reportedly been diagnosed in the preceding 2 months. The size of the residual burden has, however, remained high and could easily be underestimated if statutory reporting statistics alone is used for epidemiologic surveillance. The high vaccination uptake rate and its protective effect might have played a role in minimizing the population risk. RAT mass screening has contributed to the assessment of the epidemiologic situation in a receding Omicron wave in Hong Kong.

Our population-based survey carried some limitations, notably self-selection bias with older and health-conscious adults and those testing positive being attracted to join the survey. The uptake rate may, therefore, be overestimated. In the analysis, we have performed age-adjustment to better reflect the situation in the population. The generalization of the results to the entire Hong Kong population should be cautioned due to the use of nonprobability sampling. Similar to other population-level surveys, recall and social desirability biases were inevitable. The survey was rolled out a week after the campaign to minimize recall bias. We assured participants of the anonymous nature of the survey to ensure the accuracy of the test result reported and compliance. By including proxy participants in the household, duplicate entries from the same household may have happened. We removed entries with the same session identifier to minimize duplicate records. As multiple brands of RAT were distributed and used with different sensitivity and specificity levels, their performance was unlikely to be perfect, so even if all participants were sampled and interpreted and reported the results correctly, the true infection status of all individuals might not have been determined definitively. It should also be noted the positive predictive value could be low in places where the prevalence is low [25]. Previous studies have, however, showed that RAT had a low false-positive rate [26] but an adequate sensitivity to identify asymptomatic and high–viral load cases [27]. The 1.03% positive rate found in this study was similar to the estimated daily point-prevalence on the last day of the campaign (0.76%, 95% CI 0.32-1.56%) [28], demonstrating the reliability of the results from this population-based survey. In conjunction with its low cost, RAT is well positioned to be used should mass screening be adopted as a cost-effective intervention in the public health control of COVID-19 [29]. As a perfect reporting rate of positive results is unlikely to be achieved, an accompanying survey would be needed and could be a feasible and appropriate means to estimate the actual prevalence in the community.

### Conclusions

In a receding Omicron wave in 2022, a large proportion of residents in Hong Kong self-performed an RAT during the “voluntary universal rapid testing” campaign promoted by the government. RAT could be a useful adjunct not just for clinical diagnosis but also as a tool for public health surveillance and self-detection of infection. Accompanied with an information system, isolation facilities, and supporting services, voluntary mass RAT screening could support the estimation of the residual population burden and for supplementing risk assessment.

## Appendix

Multimedia Appendix 1 Geographical distribution of participants who self-performed a rapid antigen test (RAT) at least once during the campaign period.

## Abbreviations

aOR: adjusted odds ratio
OR: odds ratio
PCR: polymerase chain reaction
RAT: rapid antigen test
