# Supplementary figures and images for: Mass Screening of SARS-CoV-2 With Rapid Antigen Tests in a Receding Omicron Wave: Population-Based Survey for Epidemiologic Evaluation
Source: JMIR Public Health Surveill. 2022 Nov 9;8(11):e40175. doi: 10.2196/40175 (PMC9651000; doi:10.2196/40175)

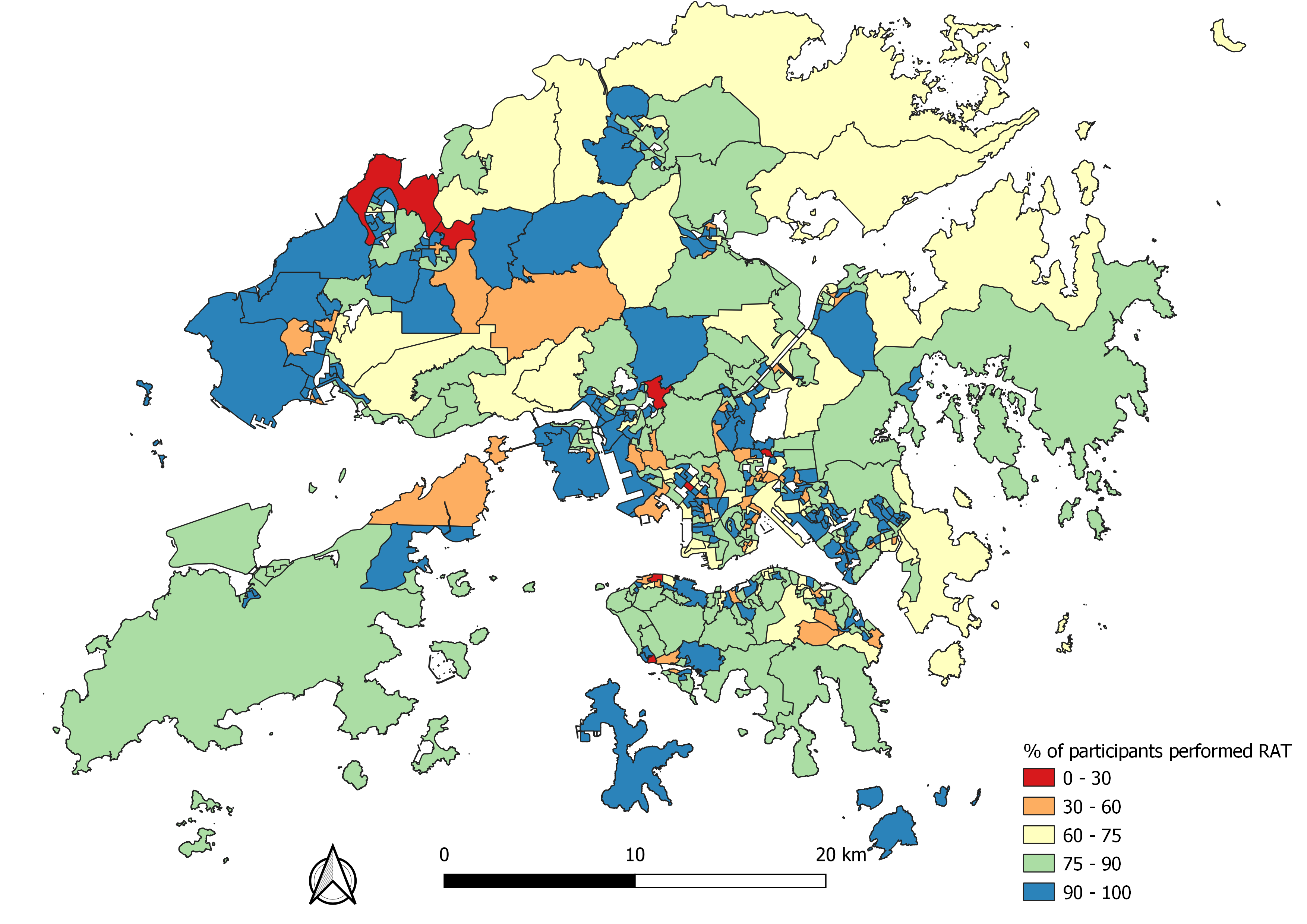

Supplement: Multimedia Appendix 1 [file publichealth_v8i11e40175_app1.png]
